# A Case of Necrotizing Fasciitis Caused by Mixed Infection of Arcanobacterium haemolyticum and Streptococcus agalactiae

**DOI:** 10.7759/cureus.85951

**Published:** 2025-06-13

**Authors:** Masaji Saijo, Shadia Constantine, Toshiaki Wakai, Naoki Wada, Mitsuo Narita

**Affiliations:** 1 Primary Center, Sapporo Tokushukai Hospital, Sapporo, JPN; 2 Family Medicine, University of Michigan, Ann Arbor, USA; 3 Infectious Disease, Sapporo Tokushukai Hospital, Sapporo, JPN; 4 Pediatrics, Sapporo Tokushukai Hospital, Sapporo, JPN

**Keywords:** arcanobacterium haemolyticum, co-infection, diabetes mellitus, immunocompromised host, necrotizing fasciitis, soft tissue infection, streptococcus agalactiae

## Abstract

*Arcanobacterium haemolyticum* (*A. haemolyticum*), a Gram-positive, anaerobic, rod-shaped bacterium, is typically associated with pharyngitis and skin infections but can also cause severe, life-threatening infections such as sepsis and necrotizing fasciitis (NF), particularly in immunocompromised patients. We report a case of severe NF of the lower extremity caused by a mixed infection with *A. haemolyticum* and *Streptococcus agalactiae* (*S. agalactiae*) in a patient with diabetes mellitus. A 51-year-old male with poorly controlled diabetes mellitus presented with extensive swelling, necrosis, and bullae formation in his left lower extremity five days after sustaining a puncture wound. Laboratory tests revealed elevated creatine kinase and inflammatory markers, and imaging identified subcutaneous gas. The patient was diagnosed with NF and underwent emergent debridement. Blood and intraoperative tissue cultures grew both *A. haemolyticum* and *S. agalactiae*. Despite aggressive surgical management and appropriate antibiotic therapy, the infection progressed, and the patient underwent a below-ankle amputation on hospital day seven and a below-knee amputation on hospital day 29. Antimicrobial therapy was concluded on day 32. This case underscores the potential for *A. haemolyticum*, particularly in combination with other pathogens such as *S. agalactiae*, to cause rapidly progressing and life-threatening NF, particularly in patients with underlying risk factors such as poorly controlled diabetes mellitus.

## Introduction

*Arcanobacterium haemolyticum* (*A. haemolyticum*) is a Gram-positive, catalase-negative, facultative anaerobic bacterium that colonizes the human pharynx and skin [1]. It is a known cause of pharyngitis, particularly in adolescents, where it may present with a scarlatiniform rash. Although commonly associated with mild upper respiratory tract infections, *A. haemolyticum* can also cause skin and soft tissue infections [2]. More recently, it has been increasingly implicated in severe invasive infections - including osteomyelitis, bacteremia, sepsis, and necrotizing fasciitis (NF) - particularly in individuals with immunocompromising conditions or chronic comorbidities [1-3].

NF is a rapidly progressive, life-threatening infection of the fascia and subcutaneous tissues associated with high morbidity and mortality. While group A streptococci and polymicrobial infections are the most common etiologies, less common pathogens such as *A. haemolyticum* may play a significant role, especially in the context of co-infection.

Here, we describe a case of NF of the lower extremity due to a mixed infection with *A. haemolyticum* and *Streptococcus agalactiae* (*S. agalactiae*) in a patient with poorly controlled diabetes mellitus.

## Case presentation

A 51-year-old male presented to the emergency department with severe pain and swelling of the left lower extremity. He reported a five-day history of progressive symptoms following a puncture wound to the sole of his left foot sustained after stepping on discarded material. His medical history was significant for longstanding hyperglycemia, which had never been treated with medications.

**Figure 1:**
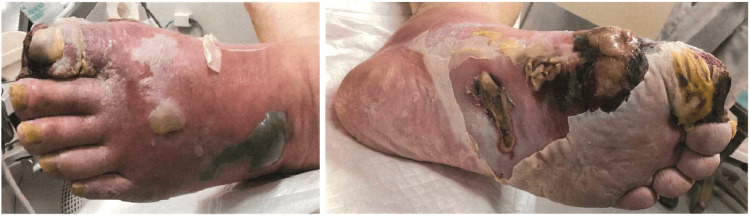
Skin changes on the left foot on admission

On arrival, vital signs were as follows: temperature of 38.0°C, heart rate of 118 beats per minute, blood pressure of 175/88 mmHg, respiratory rate of 20 breaths per minute, and oxygen saturation of 98% on room air. Physical examination revealed diffuse swelling from the foot to the thigh. The plantar aspect of the foot showed significant desquamation, erosion, and necrosis, while large pustules and hemorrhagic bullae were present on the dorsum (Figure 1).

**Figure 2:**
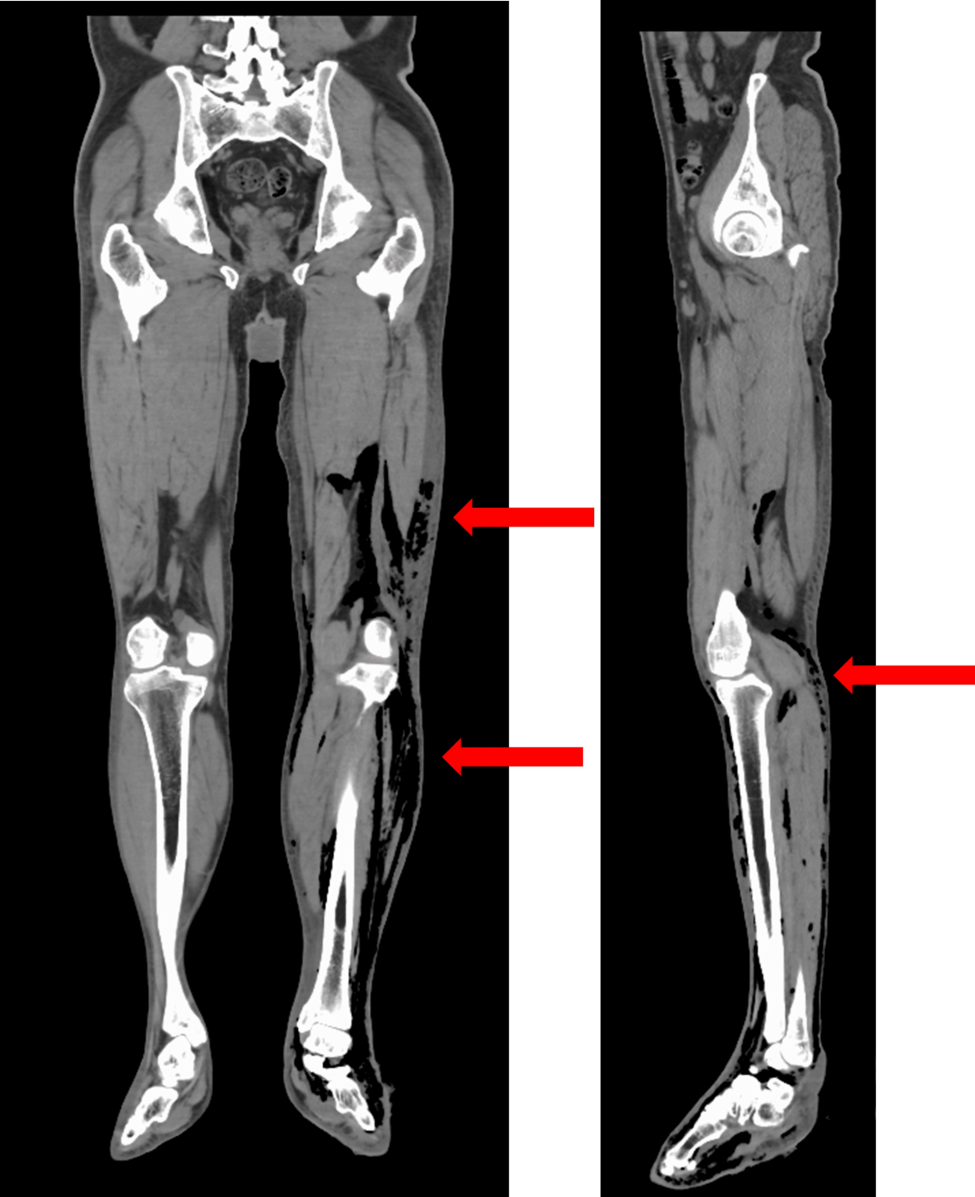
CT scan of both lower legs on the day of admission (arrow indicates subcutaneous and intermuscular air)

Blood test results obtained at admission (Table 1) demonstrated significant leukocytosis, elevated inflammatory markers, and rhabdomyolysis. Additionally, significant hyperglycemia secondary to poorly controlled diabetes was observed. Computed tomography (CT) of the lower extremity demonstrated diffuse subcutaneous edema and soft tissue gas extending from the thigh to the lower leg, suggesting necrotizing fasciitis (Figure 2).

**Table 1:** Blood test results on admission

Variables	Values	Reference range
Hemoglobin (g/dL)	11.6	13-17
White blood cell (/mm^3^)	42,800	3,300-8,600
Platelet count (/mm^3^)	398,000	150,000-350,000
Creatinine (mg/dL)	1.43	0.65-1.07
Urea (mg/dL)	36	8-20
Sodium (meq/L)	118	138-145
Potassium (meq/L)	5.1	3.6-4.8
Chloride (meq/L)	79	101-108
Bicarbonate (meq/L)	20.9	22-26
Alanine transaminase (U/L)	53	10-42
Aspartate transaminase (U/L)	67	13-30
C-reactive protein (mg/dL)	36.55	0.00-0.14
Creatine phosphokinase (U/L)	745	59-248
Myoglobin (ng/mL)	1,912	23-70
Glucose (mg/dL)	496	73-109
HbA1c (%)	11.6	4.9-6.0

The patient was taken emergently to the operating room for surgical debridement under general anesthesia. Broad-spectrum intravenous antibiotics - clindamycin and ampicillin/sulbactam - were initiated postoperatively.

Upon intensive care unit (ICU) admission, the patient presented with hyperglycemia (blood glucose of 500 mg/dL). A continuous intravenous insulin infusion was initiated, leading to a reduction in blood glucose to 200s mg/dL by hospital day three, at which point the infusion was discontinued. Subsequently, blood glucose was managed with a regimen of insulin glargine six units once daily, insulin lispro four units three times daily, and correctional insulin based on an insulin scale. Oral intake commenced on hospital day three. As glycemic control stabilized, the insulin regimen was transitioned on hospital day nine to insulin glargine eight units once daily and insulin lispro four units three times daily, which effectively maintained blood glucose levels between 130 and 200 mg/dL.

**Figure 3:**
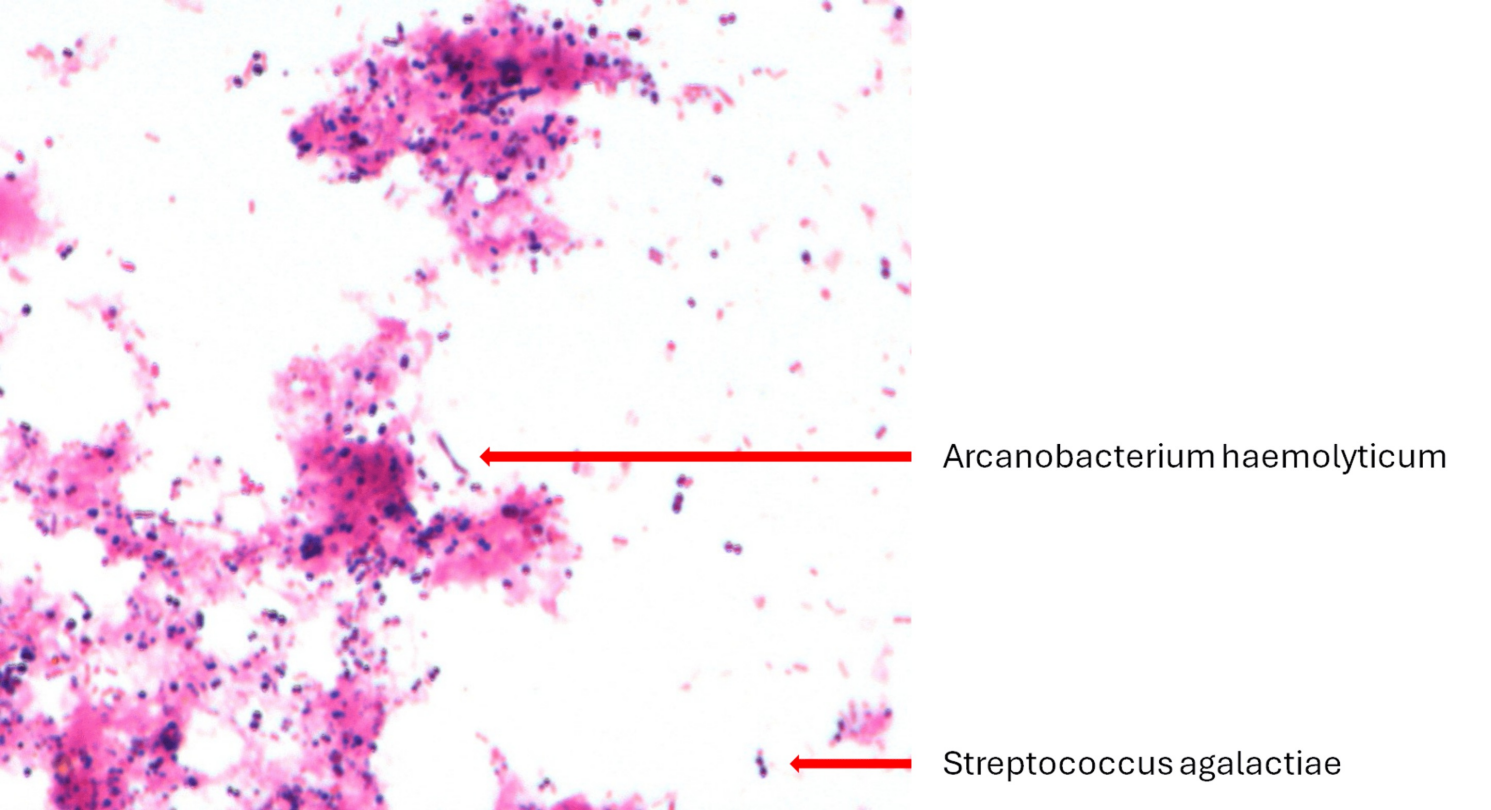
Gram stain image of cultures from tissue samples

Blood cultures taken at the time of admission were negative in both sets. Intraoperative cultures from four separate tissue samples grew *A. haemolyticum* and *S. agalactiae* (Figure 3).

A comprehensive microbiological workup confirmed the diagnosis of a mixed bacterial infection. Identification via matrix-assisted laser desorption/ionization time-of-flight mass spectrometry (MALDI-TOF MS) confirmed the presence of both *A. haemolyticum* and *S. agalactiae*.

**Figure 4:**
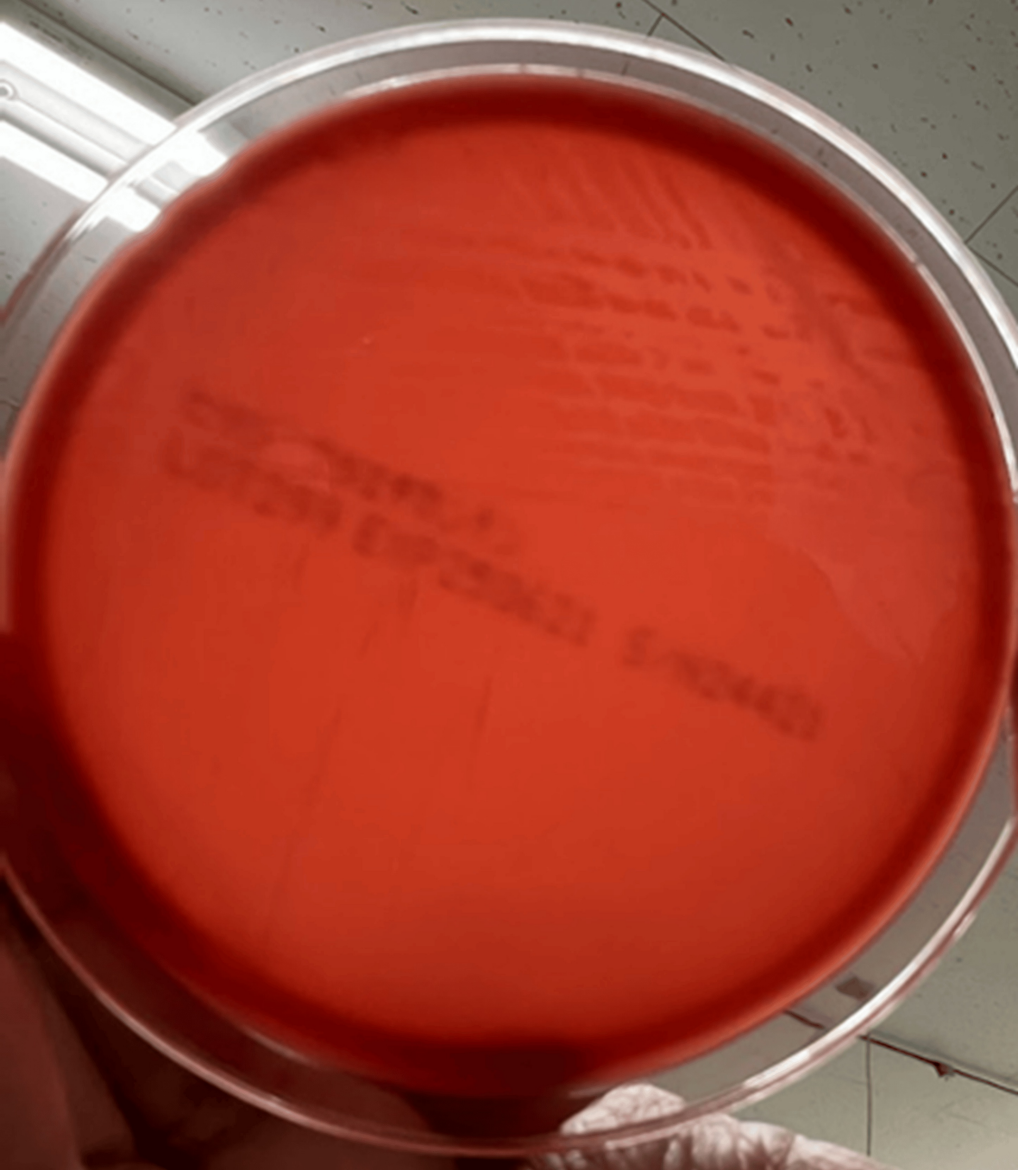
Blood agar culture findings after 24 hours of culture of the separated sample Only weak hemolysis is observed.

**Figure 5:**
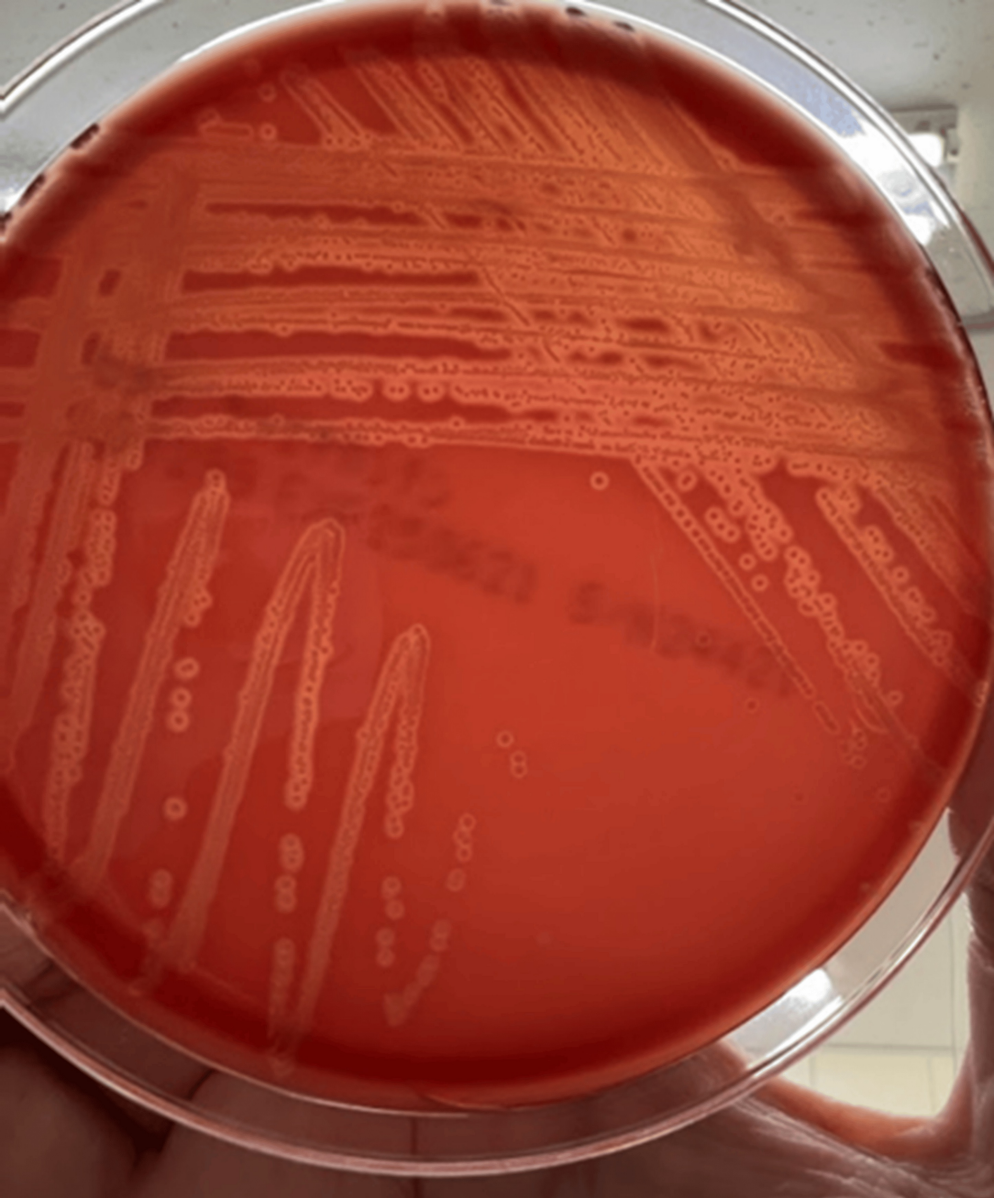
Blood agar culture findings after 48 hours of culture of the separated sample Narrow beta hemolysis rings were observed.

**Figure 6:**
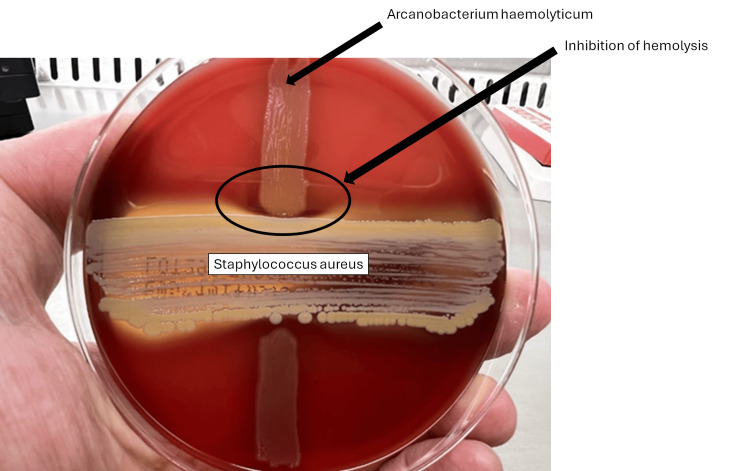
The Christie-Atkins-Munch-Peterson (CAMP) inhibition test findings Hemolysis by *S. aureus *was inhibited by *A. haemolyticum*.

**Figure 7:**
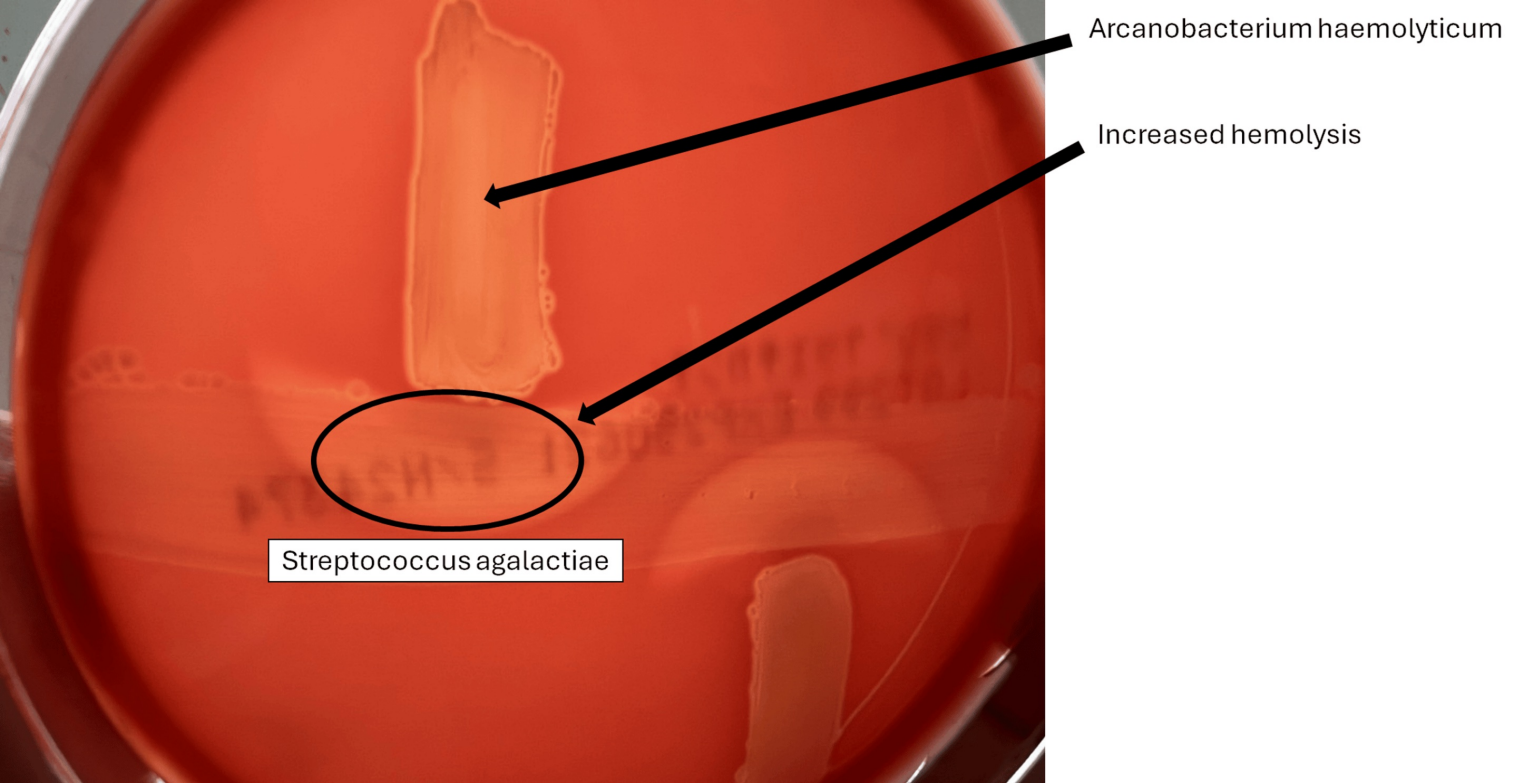
The reverse CAMP test findings Hemolysis by *S. agalactiae *was increased by *A. haemolyticum*.

As microbiological analysis, separate cultures were incubated, and small colonies were observed after 24 hours of incubation, but no beta hemolysis rings were observed (Figure 4). After 48 hours of incubation, narrow beta hemolysis rings were observed (Figure 5), which indicates its weak beta-hemolysis of *A. haemolyticum*. Further characterization using the Christie-Atkins-Munch-Peterson (CAMP) inhibition test yielded positive results (Figure 6). The reverse CAMP test also showed positive results (Figure 7). These findings support the diagnosis of *A. haemolyticum *[4].

Based on susceptibility testing (Table 1), the antibiotic regimen was narrowed to intravenous ampicillin/sulbactam monotherapy.

**Table 2:** Antibiotic susceptibility profile for the detected A. haemolyticum

Variables	MIC (μg/mL)	Susceptibility
Penicillin G	0.25	Sensitive
Cefotaxime	0.5	Sensitive
Cefepime	2	Intermediate
Ceftriaxone	0.5	Sensitive
Erythromycin	<0.12	Sensitive
Trimethoprim/sulfamethoxazole	<0.5	Sensitive
Vancomycin	0.5	Sensitive
Rifampicin	<1	Sensitive

Even with early surgical intervention and tailored antimicrobial therapy, the infection continued to progress. On hospital day seven, the patient underwent a below-ankle amputation of the left leg due to worsening necrosis. The ongoing deterioration of tissue viability ultimately necessitated a below-knee amputation on hospital day 29. Nonetheless, antimicrobial therapy was concluded on day 32, as evidenced by no subsequent fever flare-ups and no signs of infection at the amputation site [5-7].

## Discussion

This case illustrates a severe and rapidly progressive presentation of NF caused by a mixed infection with *A. haemolyticum* and *S. agalactiae* in a patient with poorly controlled diabetes mellitus.

*A. haemolyticum* is increasingly recognized as a pathogen in severe skin and soft tissue infections [2]. Although it is more commonly associated with wound infections, cases of NF due to *A. haemolyticum* have been documented, particularly in immunocompromised hosts [1,3].

Diabetes mellitus is a significant risk factor for NF, irrespective of the causative agent. This heightened risk stems from impaired immune function, microvascular damage, and neuropathy, which delay wound healing and increase susceptibility to infection. Clinical reviews and case series consistently show a high prevalence of diabetes among NF patients, with reported rates ranging from 44% to 61% [8,9]. Diabetic patients with NF also face a higher incidence of complications, including polymicrobial infections, atypical symptoms, and limb loss [8,10,11].

The co-infection of* A. haemolyticum* and *S. agalactiae* in this context is notably rare. To our knowledge, the case that Lee et al. reported is the only similar case that has been reported previously involving a diabetic patient with polymicrobial NF who also survived [12]. Their case was caused by a co-infection involving *A. haemolyticum*, *S. agalactiae*, and *Finegoldia magna*. These cases suggested that enhanced hemolytic activity might exacerbate tissue destruction in polymicrobial infections.

The initial empiric antibiotic regimen of clindamycin and ampicillin/sulbactam was appropriate for suspected polymicrobial NF. Ampicillin/sulbactam provides broad coverage against Gram-positive, Gram-negative, and anaerobic organisms. At the same time, clindamycin covers Gram-positive and anaerobic bacteria and inhibits bacterial toxin production - a beneficial property in streptococcal infections. Once cultures confirmed susceptibility, de-escalation to ampicillin/sulbactam monotherapy was a rational and evidence-based choice.

Despite prompt surgical debridement and appropriate antimicrobial therapy, the infection progressed, eventually necessitating below-ankle and later below-knee amputation. These results highlight the aggressive nature of NF in immunocompromised patients. However, they also emphasize the importance of early detection, prompt intervention, and ongoing multidisciplinary management, demonstrating that even severe cases can be survivable with proper care.

## Conclusions

We report a rare case of NF of the lower extremity caused by a mixed infection with *A. haemolyticum* and *S. agalactiae* in a patient with poorly controlled diabetes mellitus. This case highlights the need to consider *A. haemolyticum* in the differential diagnosis of rapidly progressive soft tissue infections, particularly in immunocompromised individuals. Co-infections involving *A. haemolyticum* may exacerbate tissue destruction and complicate management. Our experience suggests that, despite the aggressive nature, timely diagnosis, aggressive surgical debridement, and targeted antimicrobial therapy are vital for patient survival.
